# A New BCN Compound with Monoclinic Symmetry: First-Principle Calculations

**DOI:** 10.3390/ma15093186

**Published:** 2022-04-28

**Authors:** Zhenyang Ma, Chunzhi Tang, Chunlei Shi

**Affiliations:** 1 Key Laboratory of Civil Aircraft Airworthiness Technology, Civil Aviation University of China, Tianjin 300300, China; zyma@cauc.edu.cn (Z.M.); clshi01@126.com (C.S.); 2 College of Safety Science and Engineering, Civil Aviation University of China, Tianjin 300300, China

**Keywords:** carbon allotropes, semiconductor material, electronic properties, anisotropy mechanical properties

## Abstract

In this study, we predicted and investigated a new light-element compound B-C-N in *Pm* phase, denoted as *Pm*-BCN, using density functional theory. *Pm*-BCN is mechanically, dynamically, and thermodynamically stable. The elastic moduli of *Pm*-BCN are larger than those of other B-C-N and light-element compounds, such as *P*2_1_3 BN, B_2_C_3_, *P*4/*m* BN, *Pnc*2 BN, and dz4 BN. By studying the mechanical anisotropy of elastic moduli, we proved that *Pm*-BCN is a mechanically anisotropic material. In addition, the shear anisotropy factors *A*_2_ and *A_Ba_* of *Pm*-BCN are smaller than those of the seven B-C-N compounds mentioned in this paper. *Pm*-BCN is a semiconductor material with an indirect and wide band gap, suggesting that *Pm*-BCN can be applied in microelectronic devices.

## 1. Introduction

Designing new light-element atoms based on boron, carbon, and nitrogen, which easily form strong covalent bonds to form compounds, is an important method of finding new multifunctional materials. New theoretically proposed materials include superhard materials [1,2,3,4,5,6,7,8,9,10,11,12,13,14,15,16], direct bandgap materials [17,18], hydrogen and lithium storage materials [19,20], and metal materials that can be used for the preparation of battery cathode materials [1,4,21,22].

Three new carbon allotropes with an orthogonal structure, *o*P-C_16_, *o*P-C_20_, and *o*P-C_24_, were proposed based on first-principles calculations [1], all of which showed metallicity. The hardness of *o*P-C_16_, *o*P-C_20_, and *o*P-C_24_ are 47.5, 49.6, and 55.3 GPa, respectively. The ideal shear strengths of *o*P-C_16_, *o*P-C_20_, and *o*P-C_24_ are higher than those of Cu and Fe, and Al. Yu et al. [23] predicted and studied a new *sp*^2^ hybrid BN polymorph, *Pnc*2 BN, which showed mechanical and dynamic stability, and found that the elastic properties of *Pnc*2 BN are better than those of dz4 BN. The indirect band gap of *Pnc*2 BN calculated using the Heyd–Scuseria–Ernzerhof (HSE06) functional is 3.543 eV, indicating that *Pnc*2 BN has semiconductor properties. On the basis of density functional theory (DFT) calculations [24,25], *m*-B_3_CN_3_ and *m*-B_2_C_3_N_2_, two new superhard BCN compounds, were designed by Xing et al. [5]. The shear modulus *B*, bulk modulus *G*, and Young’s modulus *E* of *m*-B_3_CN_3_ and *m*-B_2_C_3_N_2_ are 345, 778, and 346, respectively; the *B*, *G*, and *E* for *m*-B_3_CN_3_ and *m*-B_2_C_3_N_2_ are little bit larger than those of *o*-BC_6_N [2], *t*-BC_6_N-1 [2], and *t*-BC_6_N-2 [2]. Both *m*-B_3_CN_3_ and *m*-B_2_C_3_N_2_ are superhard materials because both compounds have a hardness in excess of 40 GPa. The structural properties, anisotropy characteristics, elastic characteristics, and electronic properties, as well as the stability, of *P*4/*m* BN were investigated by Yu et al. [26]. By adopting DFT, Xing et al. established and studied CN and BCN_2_ compounds with superhard characteristics and a space group of *C*2/*m* [4]. The hardness of CN is 58.63 GPa, and it is a semiconductor material, whereas BCN_2_ is metallic. A superhard material, *t*-C_8_B_2_N_2_, was designed by Zhu et al. [10] and Wang et al. [11]. The bulk modulus of *t*-C_8_B_2_N_2_ was found to be 383.4 [10] and 383.0 GPa [11], and the hardness was 64.7 [10] and 63.2 GPa [11].

In this study, we predicted a BCN polymorph, *Pm* BCN, which is mechanically and dynamically stable. We analyzed the structural, mechanical, and electronic characteristics of *Pm* BCN through first-principles calculations.

## 2. Theoretical Methods

On the basis of DFT calculations [24,25], we proposed and investigated a new light-element compound using the Cambridge Serial Total Energy Package (CASTEP) [27]. We adopted the generalized gradient approximation (GGA) with the Perdew–Burke–Ernzerhof (PBE) [28] and local density approximation (LDA) [29] functionals to describe the exchange and correlation potentials. To ensure that the crystal structure of *Pm*-BCN was optimal, we used Broyden–Fletcher–Goldfarb–Shanno (BFGS) [30] for geometry optimization. The convergence accuracy during optimization was less than 0.001 eV. We described the valence electrons by ultrasoft pseudopotentials [31]. We adopted the Monkhorst–Pack *k*-points for the *k*-points separation of 6 × 16 × 7 and found that the plane wave cut-off energy *E*_cut-off_ is 500 eV for *Pm*-BCN. For the phonon spectra of *Pm*-BCN, we used the density functional perturbation theory (DFPT) approach [32], and for the electronic band structures of *Pm*-BCN, we adopted the HSE06 hybrid functional [33]. In addition, we used the Voigt–Reuss–Hill (VRH) approximations [34,35,36] to calculate the bulk modulus and shear modulus.

## 3. Results and Discussion

**Figure 1:**
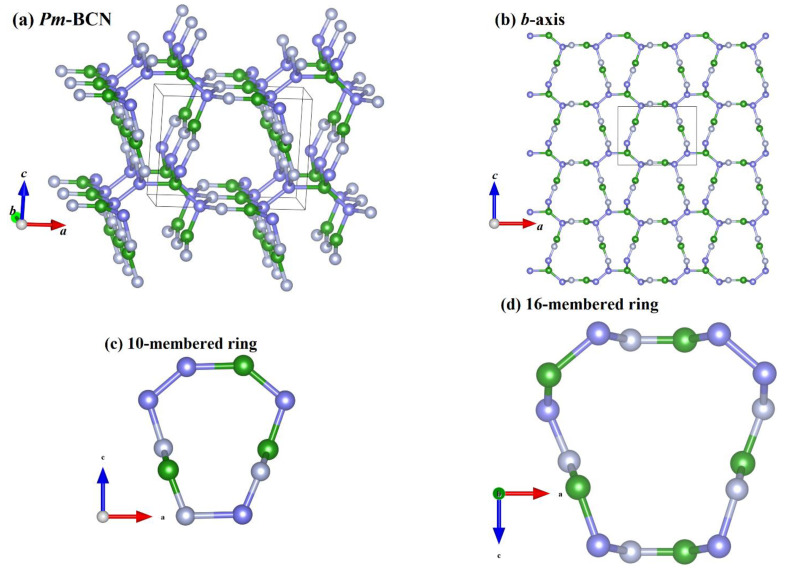
Crystal structures of *Pm*-BCN (**a**), Crystal structures of *Pm*-BCN along *b*-axis (**b**), the 10-membered ring structure (**c**), and the 16-membered ring structure (**d**).

The crystal structure of *Pm*-BCN and its structure along the *b*-axis are shown in Figure 1a,b, respectively. Blue, gray, and purple spheres represent the B, N, and C atoms, respectively. In addition to the common rings such as the 4- and 6-membered rings in the crystal structures of *Pm*-BCN, two larger rings, a 10- and a 16-membered ring, are present in the crystal structure, the structures of which are depicted in Figure 1c,d. The 4-membered ring consists of one B, one N, and two C atoms; the 6-membered ring consists of one B, one N, and four C atoms; the 10-membered ring consists of three B, three N, and four C atoms. The 16-membered ring consists of five B, five N, and six C atoms. The conventional *Pm*-BCN cell contains 12 atoms. Because *Pm*-BCN has a monoclinic system, the crystal structure of *Pm*-BCN is not symmetrical, and the position of each atom is different. Boron atoms occupy four positions: B1 1*a* (0.11803, 0.00000, and 0.20845), B3 1*a* (0.22107, 0.00000, and 0.73817), B10 1*b* (0.80293, 0.50000, and 0.62435), and B12 1*b* (0.59805, 0.50000, and 0.04251); nitrogen atoms occupy four positions: N2 1*a* (0.78254, 0.00000, and 0.74717), N7 1*b* (0.18971, 0.50000, and 0.61515), N8 1*b* (0.87536, 0.50000, and 0.36710), and N11 1*b* (0.40657, 0.50000, and 0.04285); and carbon atoms occupy four positions: C4 1*a* (0.88003, 0.00000, and 0.20041), C5 1*a* (0.71892, 0.00000, and 0.01576), C6 1*a* (0.29294, 0.00000, and 0.01183), and C9 1*b* (0.11385, 0.50000, and 0.37443). Table 1 shows the crystal lattice parameters of the B-C-N compounds. The crystal lattice parameters of *Imm*2 BCN and *I-*4*m*2 BCN are close to those previously reported [14]; therefore, the crystal lattice parameters of *Pm*-BCN reported in this manuscript are both convincing and reliable.

**Figure 2:**
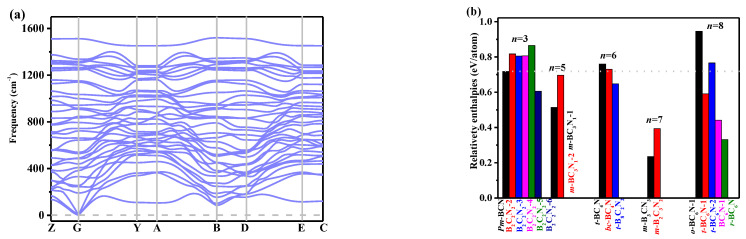
Phonon spectra of *Pm*-BCN (**a**) and the relative enthalpies of B-C-N compounds (**b**).

The stability of *Pm*-BCN through phonon spectra (Figure 2a), relative enthalpy (Figure 2b), and elastic parameters. In Figure 2a, no curve appears below zero, so *Pm*-BCN is dynamically stable. We calculated the formation energies of B-C-N compounds as: Δ*H* = *H*_B*x*C*y*N*z*_/*m*-*xH_c-_*_BN_/*n*-*yH*_diamond_/*p*. As several B-C-N compounds have an equal number of nitrogen and boron atoms, here, *x* is equal to *z*; *m*, *n*, and *p* are the B*_x_*C*_y_*N*_z_* unit and atom numbers of the conventional cell for B-C-N compounds, c-BN, and diamond. The formation energy of *Pm*-BCN is 0.7182 eV/atom, which is slightly lower than those of *o*-BC_6_N-1, *t*-BC_6_N-2 [2], B_2_C_2_N_2_-2, B_2_C_2_N_2_-3, B_2_C_2_N_2_-4, and B_2_C_2_N_2_-5 [38]. We found that the *Pm*-BCN is a metastable phase. Notably, B-C-N compounds with positive formation energies are not unusual [2,4,5,7,8,38].

For the monoclinic structure, the Born mechanical stability conditions are [39]: *C*_11_ > 0, *C*_22_ > 0, *C*_33_ > 0, *C*_44_ > 0, *C*_55_ > 0, *C*_66_ > 0, [*C*_11_ + *C*_22_ + *C*_33_ + 2 (*C*_12_ + *C*_13_ + *C*_23_)] > 0, (*C*_33_*C*_55_ − $C_{35}^{2}$) > 0, (*C*_44_*C*_66_ − $C_{46}^{2}$) > 0, (*C*_22_ + *C*_33_ − 2*C*_23_) > 0, [*C*_22_ (*C*_33_*C*_55_ − $C_{35}^{2}$) + 2 *C*_23_*C*_25_*C*_35_ − $C_{23}^{2}$*C*_55_ − $C_{25}^{2}$*C*_33_] > 0, {2[*C*_15_*C*_25_ (*C*_33_*C*_12_ − *C*_13_ *C*_23_) + *C*_15_*C*_35_(*C*_22_*C*_13_ − *C*_12_*C*_23_) + *C*_25_*C*_35_ (*C*_11_*C*_23_ − *C*_12_*C*_13_)] − [$C_{15}^{2}$(*C*_22_*C*_33_ − $C_{23}^{2}$) + $C_{25}^{2}$(*C*_11_*C*_33_ − $C_{13}^{2}$) + $C_{35}^{2}$(*C*_11_*C*_22_ − $C_{12}^{2}$)] + *C*_55_*B*} > 0, and *B* = *C*_11_*C*_22_*C*_33_ − *C*_11_$C_{23}^{2}$ − *C*_22_$C_{13}^{2}$ − *C*_33_$C_{12}^{2}$ + 2*C*_12_*C*_13_*C*_23_. Table 2 lists the *C*_ij_ values of *Pm*-BCN and other B-C-N compounds. Table 2 shows that the elastic constants of *Pm*-BCN determined by LDA are slightly higher than those determined by GGA. All the elastic constants of *Pm*-BCN satisfy the above equation for a monoclinic system, proving that *Pm*-BCN is mechanically stable. We calculated the *G* and *B* of B-C-N compounds by using the Voigt–Reuss–Hill approximation [34,35,36]. The *B_V_*, *B_R_*, *G_V_*, and *G_R_* are given by [38]:

(1) $$B_{V}=[C_{11}+C_{22}+C_{33}+2(C_{12}+C_{13}+C_{23})]/9$$

(2) $$G_{V}=[C_{11}+C_{22}+C_{33}+3(C_{44}+C_{55}+C_{66})-(C_{12}+C_{13}+C_{23})]/15$$

(3) $$\begin{array}{l} B_{R}=\Delta [(C_{33}C_{55}-C_{35}^{2})(C_{11}+C_{22}-2C_{12})+(C_{23}C_{55}-C_{25}C_{35})(2C_{12}-2C_{11}-C_{23})+(C_{13}C_{35}-C_{15}C_{33})\\ (C_{15}-2C_{25})+(C_{13}C_{55}-C_{15}C_{35})(2C_{12}+2C_{23}-C_{13}-2C_{22})+2(C_{13}C_{25}-C_{15}C_{23})(C_{25}-C_{15})+A{]}^{-1} \end{array}$$

(4) $$\begin{array}{l} G_{R}=15\{4[(C_{33}C_{55}-C_{35}^{2})(C_{11}+C_{22}+C_{12})+(C_{23}C_{55}-C_{25}C_{35})(C_{11}-C_{12}-C_{23})+\\ (C_{13}C_{35}-C_{15}C_{33})(C_{15}+C_{25})+(C_{13}C_{55}-C_{15}C_{35})(C_{22}-C_{12}-C_{23}-C_{13})+\\ (C_{13}C_{25}-C_{15}C_{23})(C_{15}-C_{25})+A]/\Delta +3[(C/\Delta )+(C_{44}+C_{66})/(C_{44}C_{66}-C_{46}^{2})]{\}}^{-1} \end{array}$$

(5) $$A=C_{11}(C_{22}C_{55}-C_{25}^{2})-C_{12}(C_{12}C_{55}-C_{15}C_{25})+C_{15}(C_{12}C_{25}-C_{15}C_{22})+C_{25}(C_{23}C_{35}+C_{25}C_{33})$$

(6) $$C=C_{11}C_{22}C_{33}-C_{11}C_{23}^{2}-C_{22}C_{13}^{2}-C_{33}C_{12}^{2}+2C_{12}C_{13}C_{23}$$

(7) $$\begin{array}{l} \Delta =2[C_{15}C_{25}(C_{33}C_{12}-C_{13}C_{23})+C_{15}C_{35}(C_{22}C_{13}-C_{12}C_{23})+C_{25}C_{35}(C_{11}C_{23}-C_{12}C_{13})]-[C_{15}^{2}(C_{22}C_{33}-C_{23}^{2})\\ +C_{25}^{2}(C_{11}C_{33}-C_{13}^{2})+C_{35}^{2}(C_{11}C_{22}-C_{12}^{2})+(C_{11}C_{22}C_{33}-C_{11}C_{23}^{2}-C_{22}C_{13}^{2}-C_{33}C_{12}^{2}+2C_{12}C_{13}C_{23})C_{55}] \end{array}$$

(8) $$B=(B_{V}+B_{R}),G=(G_{V}+G_{R})$$

Young’s modulus *E* is calculated by *E* = 9*BG*/(3*B* + *G*), and Table 2 lists the calculated elastic moduli of B-C-N compounds. The elastic moduli of *Pm*-BCN are less than those of other B-C-N compounds, and larger than those of other light element compounds, such as *Pnc*2 BN [23], *P*4/*m* BN [26], *P*2_1_3 BN [40], B_2_C_3_ [41], dz4 BN [42], etc.

**Figure 3:**
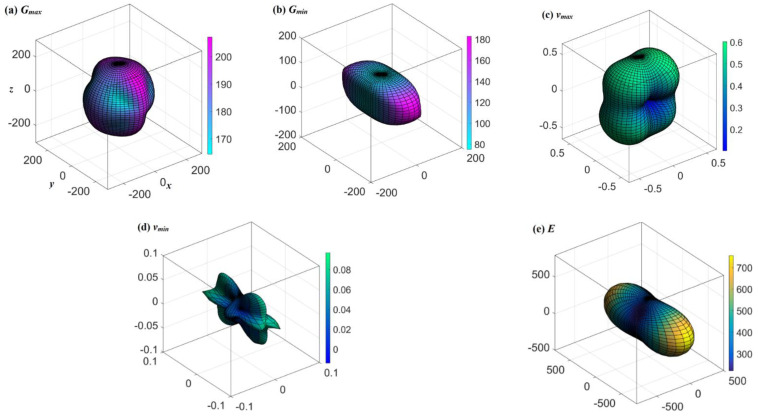
Three-dimensional structure of *G*_max_ (**a**), *G*_min_ (**b**), Poisson’s ratio *v*_max_ (**c**), *v*_min_ (**d**), and *E* (**e**) of *Pm*-BCN.

According the ElAM codes [43], we investigated the anisotropic elastic properties of *Pm*-BCN. The *G*, *v*, and *E* are illustrated in Figure 3a–e, respectively. The three-dimensional (3D) graphics of *G*, *v*, and *E* of *Pm*-BCN are not regular spheres, as shown in Figure 3. If a material possesses isotropic properties, its 3D diagram should be a regular sphere, and any shape deviating from a sphere indicates anisotropy [44,45,46,47,48,49,50]. So, we found that the *G*, *v*, and *E* of *Pm*-BCN exhibit anisotropic elastic properties. The *G*_max_/*G*_min_ and *E*_max_/*E*_min_ ratios are used to characterize the anisotropic elastic properties of *G* and *E*, which are 207.12/75.11 = 2.76 and 755.31/221.89 = 3.40 for *Pm*-BCN, respectively. As shown by the *G*_max_/*G*_min_ and *E*_max_/*E*_min_ ratios, the anisotropic elastic properties of *Pm*-BCN show that it has a greater shear modulus than B_2_C_3_N_2_ and B_2_CN_2_ [5], but a smaller one than BCN_2_ [4]. BCN_2_ has the largest Young’s modulus among *Pm*-BCN, B_2_C_3_N_2_, and B_2_CN_2_; B_2_C_3_N_2_ shows the weakest anisotropy in *E*.

**Figure 4:**
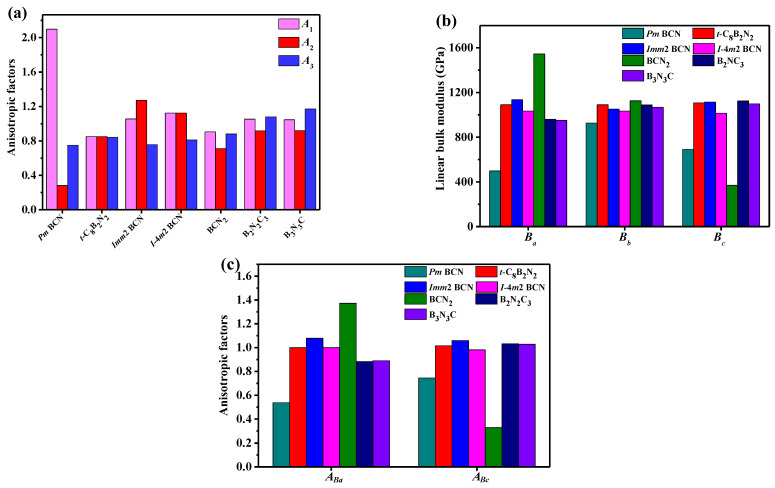
Anisotropy factor *A*_1_, *A*_2_, and *A*_3_ (**a**); linear bulk modulus *B_a_*, *B_b_*, and *B_c_* (**b**); *A_Ba_* and *A_Bc_* (**c**) for *Pm*-BCN.

For mechanical anisotropy in *G*, the shear anisotropy factor is an index of the mechanical anisotropy of atomic bonding in different shear planes. *A*_1_, *A*_2_, and *A*_3_ represent the shear anisotropic factor for the (100) shear plane between [011] and [010] directions, the (010) shear plane between [101] and [001] directions, and the (001) shear plane between [110] and [010] directions, respectively. A1 = 4C44/(C11 + C33 − 2C13), *A*_1_ = 4*C*_55_/(*C*_22_ + *C*_33_ − 2*C*_23_), *A*_3_ = 4*C*_66_/(*C*_11_ + *C*_22_ − 2*C*_11_) [51,52]. The *A*_1_, *A*_2_, and *A*_3_ of carbon allotropes of seven B-C-N compounds are illustrated in Figure 4a. We found that the *A*_1_, *A*_2_, and *A*_3_ of isotropic materials should be one; however, as shown in Figure 4a, the *A*_1_ of *Pm*-BCN is much greater than one, whereas the *A*_2_ of *Pm*-BCN is much lower than one, so *Pm*-BCN exhibits a larger anisotropy at the (100) and (010) shear plane. Among these seven B-C-N compounds, the (100), (010), and (001) shear planes of *t*-C_8_B_2_N_2_ show minimal differences, implying that the anisotropies at the three planes of the shear modulus of *t*-C_8_B_2_N_2_ are similar. The *B* along the *a*, *b*, and *c* axes, *B_a_*, *B_b_*, and *B_c_*, were calculated as [51,53]: *B_a_* = *Λ*/(1 + *α* + *β*), *B_b_* = *B_a_*/*α*, and *B_c_* = *B_a_*/*β*, and *Λ* = *C*_11_ + 2*C*_12_ + *C*_22_*α*^2^ + 2*C*_13_*β* + *C*_33_*β*^2^ + 2*C*_23_*αβ*, *α* = [(*C*_11_ − *C*_12_)(*C*_33_ − *C*_13_) − (*C*_23_ − *C*_13_)(*C*_11_ − *C*_13_)]/[(*C*_33_ − *C*_13_) (*C*_22_ − *C*_12_) − (*C*_13_ − *C*_23_)(*C*_11_ − *C*_13_)], and *β =* [(*C*_22_ − *C*_12_)(*C*_11_ − *C*_13_) − (*C*_11_ − *C*_12_)(*C*_23_ − *C*_12_)]/[(*C*_22_ − *C*_12_)(*C*_33_ − *C*_13_) − (*C*_12_ − *C*_23_) (*C*_13_ − *C*_23_)]. The anisotropy of the bulk moduli along the *a* and *c* directions with respect to the *b* directions are described by: *A_Ba_* = *B_a_*/*B_b_*, *A_Bc_* = *B_c_*/*B_b_*. Figure 4b,c shows the *B_a_*, *B_c_*, *A_Ba_*, and *A_Bc_* of the seven B-C-N compounds. The *B_a_* and *B_c_* of the seven B-C-N compounds differ. The *B_a_* of BCN_2_ is the largest, and *Pm*-BCN exhibits the smallest linear bulk modulus *B_a_*. Although BCN_2_ has the largest linear bulk modulus *B_a_*, its linear bulk modulus *B_c_* is the smallest. Figure 4c shows that the anisotropy of *B* along the *a* direction, *A_Ba_*, of *t*-C_8_B_2_N_2_ and *I*-4*m*2 BCN, and the *A_Bc_* of B_2_N_2_C_3_ and B_3_N_3_C are very close to one, indicating that the *B* of these materials is less anisotropic along the *a* and *c* axes. Additionally, *Pm*-BCN exhibits the largest anisotropy in *A_Ba_*, and BCN_2_ has the largest anisotropy in *A_Bc_*.

**Figure 5:**
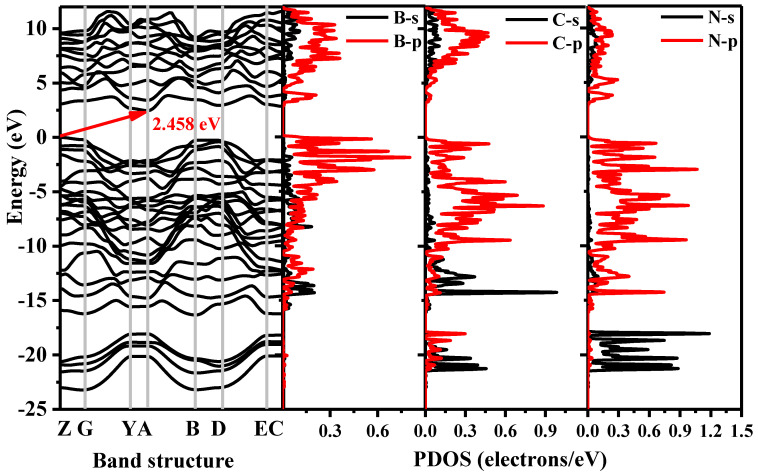
Band structure and the partial density of states (PDOS) of *Pm*-BCN.

**Figure 6:**
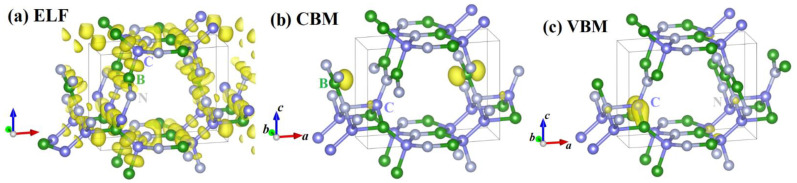
Electronic localization functions (**a**) and band decomposed charge densities of VBM and CBM (**b**,**c**) of *Pm*-BCN.

The electronic band structure and the PDOS of *Pm*-BCN obtained by the HSE06 function are shown in Figure 5, where the dashed line of zero energy (0 eV) indicates the Fermi level (*E*_F_). The valence band maximum (VBM) of *Pm*-BCN is located at Z (0.0, 0.0, 0.5), and its conduction band minimum (CBM) appears at A (0.5, 0.5, 0.0). *Pm*-BCN has an indirect and wide band gap of 2.458 eV, therefore it is clearly a semiconductor. The PDOS can be divided into three parts: the first region ranges from −23 to −18 eV, the second region ranges from approximately −16 eV to the Fermi level, and the third region ranges approximately from 2.5 to 10 eV. The first region is dominated from the *p* orbital, which is primarily from the N-s, C-s, and C-p orbitals. The N-p state and C-s orbitals provide a major contribution to the −16 to −12 eV of the second region. From −12 eV to the Fermi level, the distributions of the B-p, C-p, and N-p orbitals are much greater than that of s orbitals. From 2.5 to 10 eV, the distribution of N-p orbitals is slightly smaller than that of B-p and C-p orbitals. To further understand the chemical bonds, Figure 6 plots the electron localization function (ELF) of *Pm*-BCN. ELF is an excellent measure of the strength of covalent bonds. Here, B-C and B-N bonding are strongly covalent, whereas the C-N bonding is weakly covalent. The band decomposed charge densities of VBM and CBM of *Pm*-BCN are depicted in Figure 6b,c, respectively. The B atom is the main contributor to the CBM; the C atom contributes a small amount to the CBM but is the main contributor to the VBM; and the N atom makes a small contribution to the VBM.

## 4. Conclusions

Based on DFT calculations, in this study, we designed and predicted a new light-element compound, *Pm*-BCN. First, by analyzing the phonon spectrum, we found that the elastic constants and relative enthalpy of *Pm*-BCN are theoretically stable. Second, we found that *Pm*-BCN has an indirect and wide band gap and is a semiconductor material. Third, we showed that the B10 position is the main contributor to the CBM, the C9 position provides a small contribution to the CBM but the main contribution to the VBM, and the N8 position is a minor contributor to the VBM. Finally, we found that the elastic anisotropy in *E* and the *G* of *Pm*-BCN are slightly smaller than those of BCN_2_ according to *E*_max_/*E*_min_ and *G*_max_/*G*_min_, whereas the shear anisotropy factor *A*_2_ and the anisotropy of *B* along the *a* direction with respect to the *b* direction *A_Ba_* of *Pm*-BCN are smaller than those of *t*-C_8_B_2_N_2_, *I*-4*m*2 BCN, *Imm*2 BCN, B_2_N_2_C_3_, BNC_2_, and B_3_N_3_C.

## Figures and Tables

**Table 1:** Crystal lattice parameters of *Pm* BCN and other B-C-N compounds.

		*a*	*b*	*c*	*β*	*V*	*ρ*
*Pm* BCN	GGA	7.2538	2.5387	5.4260	89.960	24.981	2.448
	LDA	7.1697	2.5043	5.3334	89.219	23.938	2.555
*t*-C_8_B_2_N_2_	GGA ^a^	2.5470		10.9470		17.781	3.402
	LDA ^b^	2.5250		10.8540		17.092	3.539
*Imm*2 BCN	GGA	2.5451	2.5658	10.9077		17.808	3.434
	GGA ^c^	2.5453	2.5658	10.9169		17.822	
	GGA ^d^	2.5480	2.5690	10.9130		17.859	
	LDA	2.5129	2.5313	10.7687		17.125	3.571
	LDA ^c^	2.5127	2.5309	10.7659		17.212	
*I*-4*m*2 BCN	GGA	2.5648		10.9948		18.081	3.382
	GGA ^c^	2.5641		10.9892		18.063	
	GGA ^d^	2.5670		11.0020		18.124	
	LDA	2.5301		10.8420		17.101	3.525
	LDA ^c^	2.5298		10.8396		17.343	

^a^ [11], ^b^ [10], ^c^ [14], ^d^ [37].

**Table 2:** Calculated elastic constants (GPa) and elastic moduli (GPa) of *Pbca* XN, *Imm*2 BCN, and *I*-4*m*2 BCN.

		*C*_11_	*C*_12_	*C*_13_	*C*_15_	*C*_22_	*C*_23_	*C*_25_	*C*_33_	*C*_35_	*C*_44_	*C*_46_	*C*_55_	*C*_66_	*B*	*G*	*E*
*Pm* BCN	GGA	339	50	184	8	770	48	0.4	400	10	194	8	75	189	225	153	374
	LDA	364	61	212	16	829	59	2	405	20	203	12	73	199	245	154	382
*m*-B_2_C_3_N_2_	GGA ^a^	723	97	178	11	936	43	7	871	21	326	−2	394	395	351	367	816
*m*-B_3_CN_3_	GGA ^a^	684	123	181	21	841	47	−7	841	63	304	2	375	387	345	346	778
*t-*C_8_ B_2_N_2_	GGA ^b^	987	39	143					890		368			389	390	379	858
*Imm*2 BCN	GGA	963	23	144		898	141		395		395		457	343	367	394	
	GGA ^c^	962	22	143		894	140		819		400		456	343	365	394	839
*I*-4*m*2 BCN	GGA	853	45	133					753		377			327	342	358	
	GGA ^c^	857	47	135					755		377			328	345	358	798

^a^ [5], ^b^ [10], ^c^ [14].

## Data Availability

Data is contained within the article.
